# What supports applicants’ reasons for becoming physicians: a thematic analysis of their beliefs and statements

**DOI:** 10.5116/ijme.68f3.9b51

**Published:** 2025-10-30

**Authors:** Shunsuke Kimura, Fumitaka Tanemura, Satoshi Kodama, Hiroshi Nishigori

**Affiliations:** 1 Department of Cardiovascular Medicine, Machida Municipal Hospital, Machida, Japan; 2 Faculty of Human Sciences, Toyo Gakuen University, Tokyo, Japan; 3 Department of Ethics, Graduate School of Letters, Kyoto University, Kyoto, Japan; 4 Center for Medical Education, Graduate School of Medicine, Nagoya University, Nagoya, Japan

**Keywords:** Medical school applicants, admissions, reasons, lying, justification internalism

## Abstract

**Objectives:**

To address the problem of medical school applicants’ lying about their reasons for becoming physicians in the admissions process, this study aims to explore the grounds for their reasons by distinguishing between their beliefs and statements.

**Methods:**

The participants, recruited by convenience sampling, were 15 medical students and physicians who had entered graduate-entry programs of medical schools in Japan. We conducted individual semi-structured online interviews in 2020 and performed a reflexive thematic analysis.

**Results:**

We generated five themes regarding the grounds for applicants’ reasons in their beliefs: consistency with past interests, experience of being underprivileged, experience of family disease, parental influence, and no grounds; four themes regarding the grounds for applicants’ true reasons in their statements: consistency with actual past interests, actual experience of being underprivileged, actual experience of family disease, and actual experience of being powerless for patients; and four themes regarding the grounds for applicants’ untrue reasons in their statements: consistency with actual or fictional past interests, actual experience of family disease, fictional parental influence, and convenient origin.

**Conclusions:**

This study is the first to distinguish between applicants’ beliefs and statements and analyze the grounds for their reasons for becoming physicians. The findings propose a reconstruction of the concept of reasons for becoming physicians and suggest that admissions committees may be able to verify applicants’ reasons in their statements by asking them to present the grounds for them.

## Introduction

A representative question that medical school admissions committees ask applicants is why they want to become physicians. Applicants are typically required to address this question in personal statements and interviews during the admissions process.^1^^–^^5^ One of the problems related to this question is applicants’ lying about their reasons for becoming physicians. According to Kumwenda and colleagues,^6^ many applicants were aware of the option of exaggeration and dishonesty in personal statements and interviews, and a few perceived lying as a common practice in the admissions process. Moreover, White and colleagues^4^ reported applicants’ comments on personal statements: they “exaggerate but don’t get caught”; they “do whatever you have to do to get in.” Thus, applicants’ reasons for becoming physicians in their statements may differ from those in their beliefs.

This issue should be addressed from the following three perspectives. First, it could have an influence on physicians’ medical practice, in which they must be honest with their patients.^4^^,^^6^ Second, it could undermine the fairness of the admissions process because applicants who are better at such exaggeration and dishonesty may have an advantage.^4^^,^^6^ Third, it could prevent admissions committees from using applicants’ personal statements as a selection tool with sufficient validity.^7^^,^^8^ Therefore, “the ability to distinguish genuine personal statements from exaggerated accounts presents a challenge”.^6^

A systematic review by Goel and colleagues^9^ reported medical school applicants’ reasons for becoming physicians: to work for people, an interest in medicine, social status, professional growth, financial security, job security, parental wishes, work independence, working abroad or in urban areas, family experiences of disease, and family traditions. This review summarized the literature, including representative studies,^2^^,^^5^^,^^10^^–^^13^ perhaps without distinguishing between applicants’ beliefs and statements. However, McManus and colleagues^2^ claimed the importance of revealing applicants’ primary reasons in their beliefs because many might state their reasons during the admissions process, “Because I want to help people.” According to McManus and colleagues,^2^ applicants’ primary reasons for becoming physicians in their beliefs were to help people, to be indispensable, to be respected, and to be engaged in science. Other studies ^3^^–^^5^  have also analyzed applicants’ reasons for becoming physicians stated in the admissions process and indicated that applicants tend to exaggerate their reasons in their statements. Wouters and colleagues^5^ found that applicants attempted to appeal their reasons by citing their personal life events such as their illness and underprivileged stories. For example, “But the main reason why I want to become a doctor, is because I almost lost my mother when I was ten years old. […] I’ve seen what the medical community meant for my mother and me and then I knew I wanted to become a doctor”; “As a political refugee from a country where people rarely have access to basic needs such as health care, and my experience with it, at a very young age I knew that I wanted to become a doctor and that I wanted to support less fortunate people.”^5^ These statements may help us partially understand applicants’ tactics in their statements. However, it is difficult to comprehensively analyze their tactics because it is unclear whether these statements are aligned with applicants’ actual beliefs. Most prior research may have failed to consider applicants’ beliefs and statements and distinguish between their true and untrue statements. As a result, little is known about what characterizes applicants’ true and untrue reasons in their statements.

To address these knowledge gaps, we propose the concepts of lying and epistemic justification as a lens. The concept of lying has been elaborated in the field of philosophy and ethics.^14^^,^^15^ A representative definition of a lie is “a statement made by one who does not believe it with the intention that someone else shall be led to believe it.”^16^ This definition consists of four requirements.^17^ First, lying requires that one make a statement.^17^ Second, it requires that one believe that the statement is not true.^17^ Third, it requires that one make the statement to another.^17^ Fourth, it requires that one expect that another believes that the statement is true.^17^ There are two main reasons for incorporating the concept of lying as a lens. First, it can distinguish between one’s beliefs and statements. Second, it can help us understand whether one’s beliefs and statements are the same or different.

One must verify one’s belief because it may or may not be true.^18^ For example, if one believes that their reason for becoming a physician is to help people, one needs to consider whether that belief is true. The way in which one should do this has also been discussed in the field of epistemic justification.^19^ There are two ways in which one should verify one’s belief, namely justification internalism and externalism.^19^ According to justification internalism, one can approve of a belief only if one can recognize the grounds for the belief.^19^ In other words, one can see why a belief is true by reflecting on the grounds for it or “merely by sitting in one’s armchair.”^20^ For example, if one believes or states, “My reason for becoming a physician is to help people. This reason is supported by my illness experience,” it can be classified as internalism. Contrarily, justification externalism assumes that one can approve of a belief based on some reliable process even without recognizing the grounds for it.^19^ For example, if one believes or states, “My reason for becoming a physician is to help people. This reason is supported by perfect experiments about which I do not know,” it can be classified as externalism. There are two main reasons for incorporating justification internalism. First, epistemic justification is strongly related to whether one’s beliefs and statements are true. Second, we assume that justification internalism is appropriate as a lens to understand applicants’ reasons for becoming physicians because they are supposed to believe and state their reasons based on their reflections.

Based on the concepts of lying and justification internalism, reasons in applicants’ beliefs are defined as reasons that they primarily held at the time of the admissions process, and reasons in applicants’ statements are defined as reasons that they mainly presented in the admissions process. If applicants’ reasons in their statements are the same as those in their beliefs, the stated reasons are defined as true reasons; in contrast, if applicants’ reasons in their statements are different from those in their beliefs, the stated reasons are defined as untrue reasons.

In Japan, there are 82 medical schools, which consist of national, prefectural, and private schools.^21^ Medical school applicants can apply to six-year general programs after graduating from senior high school.^21^ Moreover, 29 of these 82 medical schools, most of which are national or prefectural, offer four- or five-year programs for applicants who have already acquired non-medicine bachelor’s degrees, called graduate-entry programs (GEPs).^22^ The objective of GEPs is the development of medicine by integrating it with other fields.^23^^,^^24^ The admissions process of GEPs, based on this objective, usually includes personal statements and interviews as well as academic performance. It is assumed that medical school applicants of GEPs in Japan, who must change their previous careers and present their reasons for becoming physicians in the admissions process, reflect on their reasons in advance.

This study aims to explore applicants’ reasons for becoming physicians in their beliefs and statements in terms of lying and justification internalism, using the context of GEPs of medical school in Japan. The research questions (RQs) are as follows:

     · RQ1: What supports medical school applicants’ reasons for becoming physicians in their beliefs?

     · RQ2: What supports medical school applicants’ true reasons for becoming physicians in their statements?

     · RQ3: What supports medical school applicants’ untrue reasons for becoming physicians in their statements?

## Methods

### Study design and paradigm

The research paradigm of this study was constructivism.^25^ We conducted individual semi-structured interviews and performed a reflexive thematic analysis to address the RQs.

### Setting

This study was conducted online in 2020 with successful applicants of GEPs of medical schools in Japan.

### Participants and recruitment

The participants were successful applicants of GEPs of medical schools in Japan. We did not distinguish between medical students and physicians because this study focused on participants’ beliefs and statements at the time of the admissions process. Considering recall bias, this study excluded participants who had entered GEPs over 15 years before. We assumed that it would be difficult to recruit participants because this study focused on their lying. Therefore, this study adopted convenience sampling, in which the first author (SKi) recruited his acquaintances and then requested them to introduce their acquaintances to him. We stopped recruiting participants after data saturation, which was decided after several discussions between the authors. SKi recruited 17 candidates and received consent from 15 to participate in this study. All participants provided informed consent, which included assurances of confidentiality and freedom to withdraw at any time. This study was ethically approved by the Ethics Committee of Kyoto University Graduate School and Faculty of Medicine.

### Data collection procedures

SKi conducted individual semi-structured online interviews for 60 to 120 minutes according to the interview guides. The main items of the guides were the participants’ reasons for becoming physicians in their beliefs, the grounds for these reasons in their beliefs, their reasons for becoming physicians in their statements, the grounds for these reasons in their statements, whether both reasons were the same, and why they chose to present those reasons and grounds in their statements. After the interviews, the recorded data were anonymized and transcribed into Japanese.

### Data analysis approach

We analyzed the transcribed data, assuming that “there is no single truth,” “the information being shared through the interview process is the result of an exchange between the researcher and the participant,” and “research participants and researchers are unpredictable”.^25^ This study adopted a reflexive thematic analysis to explore specific patterns and insights of the data by emphasizing the researcher’s subjectivity and interpretation.^26^^–^^28^ First, SKi read the transcribed data several times to familiarize himself with the content of the interviews. Second, SKi inductively generated initial codes from the data on the RQs. Third, SKi inductively integrated the extracted codes into broader themes. Fourth, SKi reviewed the relationship between the themes and codes and the relationship between the themes and data. Fifth, SKi defined and named the themes that were considered most representative of the data. Finally, SKi wrote the themes with the data supporting them in Japanese and translated them into English. FT and HN separately read all the transcribed data and supervised all the analyses performed by SKi. SKi, FT, and HN checked the translations by re-translating English into Japanese. The final version of the themes was identified over several discussions between the authors.

### Reflexivity

We conducted personal, interpersonal, methodological, and contextual reflexivity throughout the research process.^29^^,^^30^ This study was conducted as part of SKi’s PhD dissertation in medicine. SKi is a physician who graduated from GEPs in Japan after majoring in pharmaceutical sciences. Before graduating from the first university, SKi was repeatedly surprised to see other candidates stating their reasons for applying to a company clearly and confidently. This was because SKi had not realized what he would work for, even after careful consideration. SKi managed to enter medical school by manipulating his reason for becoming a physician in the admissions process. Therefore, SKi was interested in how applicants articulated their reasons for becoming physicians. Throughout the research process, SKi shared his personal experiences with FT and HN in advance, and all of them gradually came to understand and acknowledge the features of SKi’s original perspectives. Moreover, FT and HN advised SKi not to incorporate his perspectives excessively into this study if necessary. FT and HN supported SKi with the conceptualization and data collection and analysis of this study, as they were familiar with qualitative methods in the field of medical education. SKo supported SKi in conceptualizing this study, particularly the concepts of lying and justification internalism, as SKo was familiar with philosophy and ethics. In the interviews, SKi did not have an official position to assess the participants. After the interviews, SKi, FT, and HN reviewed them in terms of whether there were any undesirable interactions between SKi and the participants.

## Results

### RQ1

In this study, we defined reasons in medical school applicants’ beliefs as reasons that they had primarily held at the time of the admissions process. Table 1 shows the participants’ reasons for becoming physicians and the grounds for these reasons in their beliefs. All the participants held their reasons for becoming physicians in their beliefs. We generated five themes regarding what supported applicants’ reasons for becoming physicians in their beliefs.

**Table 1:** Participants’ reasons for becoming physicians and grounds for them in their beliefs

No.	Reasons for becoming physicians	Grounds for reasons for becoming physicians
1	To help people in person	Experience of being underprivileged
2	To conduct medical research	Experience of family disease
3	To earn a good salary	Consistency with past interests
4	To earn a good salary	Parental influence
5	To help people in person	Experience of being underprivileged
6	To conduct medical research	Consistency with past interests
7	To conduct medical research	Consistency with past interests
8	To earn a good salary	No grounds
9	To practice medicine	Consistency with past interests
10	To practice medicine	Experience of family disease
11	To live along with family	Experience of family disease
12	To earn a good salary	Consistency with past interests
13	To earn a good salary	Parental influence
14	To study medicine	Consistency with past interests
15	To practice medicine	No grounds

#### Consistency with past interests

Medical school applicants held their reasons for becoming physicians in their beliefs based on consistency with the subjects and fields in which they were interested.

Participant 14 became a personal trainer at a gymnasium after majoring in exercise physiology. He remained interested in physiology after graduation. While working as a personal trainer for some physicians, he became increasingly interested in medicine, which he considered a development in physiology. He decided to enter medical school to study medicine. This reason was supported in his beliefs by the consistency and development of his past core interests.

“I had several clients who were physicians, for example, a psychiatrist, a gynecologist, and a surgeon. As I talked with them, I was gradually drawn into the world of medicine. I thought that medicine sounded interesting. Because I majored in physiology, I became interested in medicine. I had never thought that I would be able to become a physician, but I became aware of the option of becoming a physician thanks to my clients. To learn more about medicine, I believed that the option would be better.” (No. 14, 30s, Male, Postgraduate Year 1)

#### Experience of being underprivileged

Medical school applicants held their reasons for becoming physicians in their beliefs based on their own or others’ underprivileged experiences.

Participant 1 became a bureaucrat in charge of education administration after majoring in law at university. As an adolescent, he was forced to leave high school due to major emotional distress and subsequently acquired a qualification to apply to university. According to him, his mission was to work for children suffering from conditions similar to those he had encountered. Although he became a bureaucrat for that mission, he felt conflicted because he was unable to resolve their personal and concrete issues. After encountering a pediatric psychiatrist by chance, he decided to become one himself to help underprivileged children in person. This reason was supported in his beliefs by his underprivileged experience.

“To tell you the truth, as an adolescent, I was mentally distressed and had to drop out of high school. After that, I managed to qualify for college admissions and enter a university. Generally, adolescence is a difficult time in life. Therefore, I hoped that I would work for children who had difficulties… At a committee meeting, I had the chance to work with a pediatric psychiatrist. There, I first learned about the field of pediatric psychiatry. Thereby, I came to realize that what I wanted to do was not to create a system to support a wide range of underprivileged children, including those who were not able to go to school for some reason, but to be able to approach individual underprivileged children because each child has their respective concrete problems. I thought that this idea underlay my reason for becoming a physician… Rather, I am always focusing on the personal problems of children. This is probably based on my experiences. In my opinion, a child does not receive value unless their personal problems are resolved. After all, bureaucrats just do their jobs as prescribed. When they finish their work at a department, they just move on to a different department. At that time, I considered that point important.” (No. 1, 30s, Male, Year 5)

#### Experience of family disease

Medical school applicants held their reasons for becoming physicians in their beliefs based on their experiences of family disease.

Participant 2 became a medical engineer in charge of dialysis at a hospital after graduating from university. While working at the hospital, his father, who resided with him, was diagnosed with mitochondrial myopathy, an intractable disease. Although he had to take care of his father, he was unable to reduce his workload because of the strict work policies of his workplace. Therefore, he decided to quit his job, after hearing about the option of entering GEPs of medical school by chance. Initially, he regarded his severe working conditions as the reason for becoming a physician. However, by looking inside himself, he realized that his “true” reason was to conduct medical research, based on the experience of his father’s disease.

“I reflected considerably on what the true reason inside of me was. Thereby, it occurred to me that my father’s disease underlay my reason for becoming a physician. My situation was severe and therefore had to be changed. I asked myself what I could do to resolve my situation and make everyone happy. The answer was to overcome the disease. I recognized my reason based on this idea.” (No. 2, 30s, Male, Year 4)

#### Parental influence

Medical school applicants held their reasons for becoming physicians in their beliefs based on the values learned from their parents or ideas inculcated by their family environment.

Participant 4 worked for two venture companies after majoring in British studies at university. He was born into an affluent family, and his father was an otorhinolaryngology practitioner. He grew up spoiled and did not take his work or life seriously. Although he worked for companies in pursuit of financial success, he was unable to perform well because of his spoiled mindset. Furthermore, he gradually suffered from mental distress due to the severe working conditions at the second company. He decided to enter medical school to earn a high salary. This reason was supported in his beliefs by ideas instilled within his family environment.

“When I was working for X [the name of the first venture company], I believed that I was doing my best. Recalling those days, however, I can hardly say that I was taking my work seriously. I have lived a wealthy life since I was a child. In other words, I was very spoiled. I was spoiled in my family, so I was not able to work hard in X. Basically, I think that I have always tended to take life lightly. This tendency applies to my current job at the hospital as well… After entering medical school, I would just have to acquire a license. Because my father was a practitioner, I always thought that I could manage my life by becoming a physician. This way of thinking was the concept of evacuation. It applied to my choice of entering GEPs as well. In my opinion, I passed the exam to seek an escape. Because I had the option of evacuation anytime like that, I was always unable to do my best as a businessman.” (No. 4, 30s, Male, Postgraduate Year 3)

#### No grounds

Medical school applicants held their reasons for becoming physicians in their beliefs despite the absence of, or without dependence on, the grounds for these reasons.

Participant 8 was a master’s student majoring in biology. Although he initially planned to pursue a PhD, he changed his mind after seeing some of his seniors unable to graduate or make money. He tried to get a job with a master’s degree to earn a good salary but failed. Finally, he decided to enter medical school. According to him, there were no grounds for his reason in his beliefs, which was to earn a good salary.

“I mainly wanted to solve my salary considerations. I thought that I would not be able to earn a good salary if I went straight into academic careers. I mainly wanted to solve my salary considerations. However, there were no grounds for this reason. I just considered academic careers difficult in terms of money… To be honest, I had few motivations to work for patients. Rather, my reason was to get a stable job and salary. I cannot think that I chose to enter medical school because I wanted to work for patients. I cannot think so.” (No. 8, 30s, Male, Postgraduate Year 6)

Participant 15 was a bachelor’s student majoring in nutrition. One day, she attended a lecture on nutrition and allergy delivered by a pediatrician. Since then, she longed to practice medicine, especially obstetrics and pediatric medicine. Therefore, she decided to enter medical school before graduating from her bachelor’s course. According to her, she did not need the grounds for her reason in her beliefs, which was to practice medicine.

“I cannot explain it. It is difficult. You do not need a reason for liking something, do you? Maybe, I did not need a reason for being interested in something either. I just longed to be a physician. I longed for it so much.” (No. 15, 30s, Female, Postgraduate Year 2)

### RQ2

In this study, we defined reasons in medical school applicants’ statements as reasons that they mainly presented in the admissions process. Table 2 shows the participants’ reasons for becoming physicians and the grounds for these reasons in their statements. All the participants presented their reasons for becoming physicians during the admissions process. Eight participants stated the same reasons as those that they held in their beliefs, and seven participants stated different reasons from those that they held in their beliefs. We generated four themes regarding what supported applicants’ true reasons in their statements. Of these four themes, three were the same as those that emerged in RQ1, and one did not appear in RQ1.

#### Consistency with actual past interests

Medical school applicants stated their true reasons for becoming physicians in the admissions process based on consistency with the actual subjects and fields in which they were interested.

Participant 14 stated his true reason for becoming a physician in the admissions process, which was to study medicine. Similar to supporting this reason in his beliefs, he presented it by citing his actual past major and interest in physiology.

“In my statements, my reason for becoming a physician was my interest in anatomy because I majored in exercise physiology at my previous university. Perhaps, I said that I chose to enter medical school because I thought that I would be able to study anatomy more broadly and deeply in the field of medicine… First, I honestly and straightforwardly presented my interest in exercise physiology, which I majored in. I then presented my genuine interest in physiology.” (No. 14, 30s, Male, Postgraduate Year 1)

**Table 2:** Participants’ reasons for becoming physicians and grounds for them in their statements

No.	Reasons for becoming physicians	Grounds for reasons for becoming physicians
1	To help people in person	Actual experience of being underprivileged
2	To conduct medical research	Actual experience of family disease
3*	To practice medicine	Actual experience of family disease
4*	To practice medicine	Fictional parental influence
5	To help people in person	Actual experience of being underprivileged
6	To conduct medical research	Consistency with actual past interests
7	To conduct medical research	Consistency with actual past interests
8*	To practice medicine	Consistency with fictional past interests
9*	To conduct medical research	Consistency with actual past interests
10	To practice medicine	Actual experience of being powerless for patients
11*	To practice medicine	Convenient origin
12*	To practice medicine	Convenient origin
13*	To practice medicine	Consistency with fictional past interests
14	To study medicine	Consistency with actual past interests
15	To practice medicine	Actual experience of being powerless for patients

Note: *Participants stated different reasons from those in their beliefs

#### Actual experience of being underprivileged

Medical school applicants stated their true reasons for becoming physicians in the admissions process based on their actual own or others’ underprivileged experiences.

Participant 1 stated his true reason for becoming a physician in the admissions process, which was to help people in person. Similar to supporting this reason in his beliefs, he presented it by citing his actual underprivileged experience.

“In the admissions process, I presented my reason exactly in the same way [as those in his beliefs].” (No. 1, 30s, Male, Year 5)

#### Actual experience of family disease

Medical school applicants stated their true reasons for becoming physicians in the admissions process based on their actual experiences of family disease.

Participant 2 stated his true reason for becoming a physician in the admissions process, which was to conduct medical research. Similar to supporting this reason in his beliefs, he presented it by citing his actual family disease.

“My reason for becoming a physician was generated from the process where I was taking care of my father. In my statements, I wrote that reason honestly and straightforwardly. Even if the interviewers asked me, I would be able to defend my reason. For I wrote it honestly… I am proud of that reason for becoming a physician.” (No. 2, 30s, Male, Year 4)

#### Actual experience of being powerless for patients

Medical school applicants stated their true reasons for becoming physicians in the admissions process based on their actual experiences in which they felt powerless for their patients.

Participant 15 stated her true reason for becoming a physician in the admissions process, which was to practice medicine. According to her, she did not need any grounds for her reason. To make this reason persuasive, however, she presented it by citing her actual experiences of being powerless for her patients while majoring in nutrition.

“As to my reason for becoming a physician, I was interested in obstetrics and gynecology. When I was majoring in nutrition, I once saw a patient who suffered from pregnancy-induced hypertension. Nutritionists cared for her in terms of nutrition. However, she soon ended up undergoing an emergency cesarean section. Furthermore, the baby weighed well below 3,000 grams due to premature delivery although I cannot recall the actual weight. Therefore, I realized that only physicians can really cure patients although nutritionists can support patients. In the admissions process, I prepared this experience to use as an episode which supports my wish to cure patients.” (No. 15, 30s, Female, Postgraduate Year 2)

### RQ3

As shown in Table 2, we generated four themes regarding what supported applicants’ untrue reasons in their statements. Of these four themes, three were the same as those that emerged in RQ1, and one did not appear in RQ1.

### Consistency with actual or fictional past interests

Medical school applicants stated their untrue reasons for becoming physicians in the admissions process based on consistency with the actual or fictional subjects and fields in which they were interested.

Participant 8 held his reason for becoming a physician in his beliefs, which was to earn a good salary, despite the absence of grounds for this reason. During the admissions process, he paid attention to the interviewers’ impressions of him. He stated a different reason, which was to practice medicine. To present this untrue reason, he attempted to imply his unreal interest in medicine by citing his actual part-time job.

“My interest in medicine was sparked when I assisted in clinical research at a hospital as a part-time job. Perhaps, I wrote this in my statements… I could not come up with any other reason for becoming a physician in my statements. Furthermore, that reason was not so unnatural… My main reason for becoming a physician was related to my failure to find a job. However, I did not find being a physician uncomfortable because I had worked at a hospital before… If I described my reason as money, interviewers would worse my impression.” (No. 8, 30s, Male, Postgraduate Year 6)

### Actual experience of family disease

Medical school applicants stated their untrue reasons for becoming physicians in the admissions process based on their actual experiences of family disease.

Participant 3 became a businessman at an electric power company after graduating from a master’s program in mechanical engineering. Although he focused on career and salary success, his career plans went awry when the company for which he worked was damaged in a massive earthquake. To earn a good salary, he decided to leave the company and enter medical school. He held this reason in his beliefs based on consistency of the subjects and fields in which he was interested. During the admissions process, he paid attention to the interviewers’ assessment of him. He stated a different reason, which was to practice medicine. He presented this untrue reason by citing his actual experiences of family disease.

“To be honest, I cannot perfectly recall my reason for becoming a physician in the admissions process. Perhaps, I presented a reason different from what I really believed. A relative of mine was suffering from a disease and a physician was curing them. I cited this physician. My grandfather and uncle were both physicians. I once considered going to medical school when I was in high school. Finally, however, I chose to major in engineering and work in the engineering field because I liked physics and mathematics. After leaving my previous job, I wondered what I should choose as a profession. That was when I encountered that physician. Therefore, my wish to become a physician like that physician was reawakened. I decided to enter medical school. Probably, this was my answer in the admissions process… I imagined what type of applicants I would want if I were an interviewer. I attempted to prepare the answers that such applicants were supposed to state.” (No. 3, 30s, Male, Postgraduate Year 5)

### Fictional parental influence

Medical school applicants stated their untrue reasons for becoming physicians in the admissions process based on values that they did not actually learn from their parents.

Participant 4 held his reason for becoming a physician in his beliefs, which was to earn a good salary, based on an idea instilled within his family environment. During the admissions process, he stated a different reason, which was to practice medicine. He presented this untrue reason by citing values that he did not actually learn from his father.

“Regarding the interviews, I did not need to prepare for the admissions process because I had experience in human resources and recruitment at the company. Moreover, I had experienced my own job search and career change… I explained my reason as follows in the interviews. As a child, I was unable to understand the virtues of practitioners such as my father. However, I came to understand why he continued to care for patients in one area for decades because I learned about society as a businessman. This story is easy to understand, isn’t it? It was a kind of settlement with my father. It was not a settlement of a quarrel but that of values. Before, I was unable to understand why practitioners would work for patients in one clinic every day. However, once I worked hard in Tokyo to seek a stimulus, I realized the virtues of practitioners engaged in one area for decades. To be honest, however, I do not believe that from the bottom of my heart.” (No. 4, 30s, Male, Postgraduate Year 3)

### Convenient origin

Medical school applicants stated their untrue reasons for becoming physicians in the admissions process based on the origins that were convenient for inferring these reasons.

Participant 12 was an undergraduate student majoring in veterinary medicine at university. Although he focused on salary success, he was unable to find a practical plan to earn a good salary after graduating from the course. Therefore, he decided to enter medical school. He held this reason in his beliefs based on consistency with the subjects and fields in which he was interested. During the admissions process, he attempted to present a different reason, which was to practice medicine, but failed repeatedly due to his inability to defend this reason logically. He learned that he was able to succeed in defending it by preparing a convenient origin: “for the people.” According to him, applicants could defend their reasons for becoming physicians based on such convenient origins in the admissions process. On the other hand, there was a risk of weakening the persuasiveness of their reasons unless they prepared further grounds to support these origins, such as personal illness or illness of family members.

“I strongly presented my vision ‘for the people’ because I applied to medical school. Or I mentioned that I wanted to work ‘for the people’… The interviewers often asked me why I did not select veterinary medicine. I managed to defend my reason by continuing to present the phrase ‘for the people.’ Since this phrase was persuasive, I managed to prevent a logical fallacy and mental unrest in the interviews… I had no further grounds for that phrase, did I? I wondered if I had concrete episodes for it. However, I had no episodes, for example, an experience in which a physician cures a disease of mine or my family, maybe… If I was in trouble, I was able to start from that phrase. It is like preparing for interviews. In my opinion, it is important to prepare a core phrase in one’s mind one can start from whatever the interviewers ask. By preparing such a phrase, one can be relaxed in the interviews. That kind of technique is important.” (No. 12, 30s, Male, Postgraduate Year 8)

## Discussion

To our knowledge, this study is the first to distinguish between applicants’ beliefs and statements and analyze the grounds for their reasons for becoming physicians. Moreover, it clearly discovered applicants who lied about their reasons in the admissions process, although the possibility had been suggested.^2^^–^^6^

Applicants held their reasons for becoming physicians in their beliefs based on consistency with past interests, experiences of being underprivileged, experiences of family disease, and family influences. The findings may be aligned with the literature,^4^^,^^5^ although it is unclear whether prior research has accurately grasped applicants’ beliefs. According to Goel and colleagues,^9^ experiences of family disease and parental influences were classified as reasons. However, in this study, they were classified as grounds for reasons. As Griffin and colleagues^31^ pointed out, it could be difficult to regard “because my parents want me to be a doctor” as applicants’ reason for becoming physicians. Thus, these findings may help us reconstruct the concept of reasons for becoming physicians by highlighting the distinction between reasons and grounds for them. To understand applicants who do not hold grounds for their reasons in their beliefs, one hypothesis might be that applicants cannot articulate their reasons based on any grounds because each ground requires further grounds for justification.^32^

When applicants stated their true reasons for becoming physicians in the admissions process, they presented the grounds: consistency with actual past interests, actual experiences of being underprivileged, actual experiences of family disease, and actual experiences of being powerless for patients. The findings may be aligned with the literature,^4^^,^^5^ although it is unclear whether prior research has described applicants’ true reasons. As previous studies ^3^^–^^6^  have pointed out, applicants could attempt to make their statements “ideal” to meet the expectations of admissions committees. When applicants stated their untrue reasons for becoming physicians, they presented the grounds: consistency with actual or fictional past interests, actual experiences of family disease, fictional family influences, and convenient origins. To support their untrue reasons, applicants could prepare some actual and fictional grounds for them, regardless of the presence of causation. Applicants may present their untrue reasons and grounds in the admissions process due to “privileged access,” in which only one knows what one is thinking.^19^ Furthermore, if applicants cannot articulate their reasons based on any grounds in their beliefs, they might take advantage of the arbitrariness of their reasons in their statements.

To verify whether applicants’ reasons in their statements are the same as those in their beliefs, experiences of being underprivileged might be reliable because they are cited only when applicants state their true reasons. Consistency with past interests and experiences of family disease might be controversial because they are cited as the grounds for both true and untrue reasons. Convenient origins might be doubtful because they are cited only when applicants state their untrue reasons. Admissions committees might be able to detect such origins by asking applicants to present further grounds for them.

This study had a few limitations. First, it had a risk of being subject to context and sampling bias. Second, it might include participants’ recall bias and lying about their beliefs and statements during the interviews. Third, it focused on descriptive aspects of applicants’ reasons for becoming physicians, not on normative ones such as the following questions: what should they hold as reasons for becoming physicians?; what should they hold these reasons based on?; what should they state as reasons for becoming physicians?; what should they state these reasons based on?; should admissions committees assess their beliefs or statements?; and whether is applicants’ lying morally wrong?

This study provides further implications for medical education practice and research. First, it proposes a reconstruction of the concept of reasons for becoming physicians. Second, further research can explore the generalizability and transferability of the findings in other contexts. Third, admissions committees may be able to verify applicants’ reasons by asking them to present the grounds for them. Fourth, applicants who acknowledge this article can take further measures with our suggestions to prevent them from lying based on the findings. Finally, further discussions could be possible about normative aspects of applicants’ reasons for becoming physicians in their beliefs and statements and lying about these reasons according to this study.

## Conclusions

This study is the first to distinguish between applicants’ beliefs and statements and analyze the grounds for their reasons for becoming physicians. The findings propose a reconstruction of the concept of reasons for becoming physicians and suggest that admissions committees may be able to verify applicants’ reasons in their statements by asking them to present the grounds for them.

### Acknowledgments

This work was supervised by Prof. Yasuhiko Konishi, Graduate School of Medicine, Juntendo University, and Prof. Hitomi Kataoka, Graduate School of Medicine, Kyoto University.

### Conflict of Interest

The authors declare that there is no conflict of interest.
